# AI in Orthodontics: Revolutionizing Diagnostics and Treatment Planning—A Comprehensive Review

**DOI:** 10.3390/jcm13020344

**Published:** 2024-01-07

**Authors:** Natalia Kazimierczak, Wojciech Kazimierczak, Zbigniew Serafin, Paweł Nowicki, Jakub Nożewski, Joanna Janiszewska-Olszowska

**Affiliations:** 1 Kazimierczak Private Medical Practice, Dworcowa 13/u6a, 85-009 Bydgoszcz, Poland; 2 Department of Radiology and Diagnostic Imaging, Collegium Medicum, Nicolaus Copernicus University in Torun, Jagiellońska 13-15, 85-067 Bydgoszcz, Poland; 3 Department of Emeregncy Medicine, University Hospital No 2 in Bydgoszcz, Ujejskiego 75, 85-168 Bydgoszcz, Poland; 4 Department of Interdisciplinary Dentistry, Pomeranian Medical University in Szczecin, 70-111 Szczecin, Poland

**Keywords:** orthodontics, artificial intelligence, deep learning, cephalometric analysis, radiology, CBCT, skeletal age, treatment planning

## Abstract

The advent of artificial intelligence (AI) in medicine has transformed various medical specialties, including orthodontics. AI has shown promising results in enhancing the accuracy of diagnoses, treatment planning, and predicting treatment outcomes. Its usage in orthodontic practices worldwide has increased with the availability of various AI applications and tools. This review explores the principles of AI, its applications in orthodontics, and its implementation in clinical practice. A comprehensive literature review was conducted, focusing on AI applications in dental diagnostics, cephalometric evaluation, skeletal age determination, temporomandibular joint (TMJ) evaluation, decision making, and patient telemonitoring. Due to study heterogeneity, no meta-analysis was possible. AI has demonstrated high efficacy in all these areas, but variations in performance and the need for manual supervision suggest caution in clinical settings. The complexity and unpredictability of AI algorithms call for cautious implementation and regular manual validation. Continuous AI learning, proper governance, and addressing privacy and ethical concerns are crucial for successful integration into orthodontic practice.

## 1. Introduction

Artificial intelligence (AI), a term first introduced in 1955 by John McCarthy, describes the ability of machines to perform tasks that are classified as intelligent [1]. During these 70 years, there have been cycles of significant optimism associated with the development of AI, alternating with periods of failure, reductions in research funding, and pessimism [2]. The 2015 victory of AlphaGo, a Google-developed AI application, over the “GO” world champion represented a breakthrough [2]. This AI success over a human player sparked further development and interest, which was raised by the introduction of the Chat-GPT in 2022. These events served as precursors to the remarkable growth of AI applications in various fields, including everyday life and medicine [2].

AI algorithms have already proven effective in various medical specialties, surpassing the capabilities of experienced clinicians [3,4,5,6,7]. These algorithms enable the analysis, organization, visualization, and classification of healthcare data. The development of AI algorithms in medicine has gained momentum in recent years, particularly in radiology, where medical imaging accounts for approximately 85% of FDA-approved AI programs (data for 2023) [8].

In the field of diagnostic imaging, AI can be categorized into three main domains: operational AI, which enhances healthcare delivery; diagnostic AI, which aids in the interpretation of clinical images; and predictive AI, which forecasts future outcomes [9]. Currently, the primary goals of AI in diagnostic imaging are to detect and segment structures and classify pathologies [10]. AI tools can analyze images obtained from various imaging modalities, ranging from X-ray to MRI [11,12,13,14,15].

Orthodontics, with its emphasis on cephalometric analysis and pretreatment imaging, is particularly well suited for the implementation of AI. However, AI is also being utilized in orthodontics for applications beyond cephalometric analysis. The literature on the use of AI in orthodontics can be divided into five main areas: diagnosis and treatment planning, automated landmark detection and cephalometric analysis, assessment of growth and development, treatment outcome evaluation, and miscellaneous applications [16].

The number of AI companies in the healthcare industry has experienced a remarkable increase, indicating significant growth in commercial prospects for AI [9]. AI tools are no longer limited to researchers and scientists involved in research and development projects. They are now accessible through commercially available web-based products as well. In orthodontics, the adoption of AI has led to the creation of various AI-based programs, such as WeDoCeph (Audax, Ljubljana, Slovenia), WebCeph (Assemble Circle, Seoul, Republic of Korea), and CephX (ORCA Dental AI, Las Vegas, NV, USA). These systems can automatically identify cephalometric landmarks, compute angles and distances, and generate cephalometric reports with significant findings. AI programs are now easily accessible on mobile devices, making AI tools widely available and promoting equal access for all interested users. As a result, orthodontic practices and scientific researchers utilizing AI applications have notably increased. However, this accessibility has also sparked concerns about patient safety, especially when AI is used for diagnosis and treatment.

The main objectives of this article are as follows: elucidate the principles of AI, outline its applications in the diagnostic process of modern orthodontic practices, and discuss the concerns associated with the implementation of AI algorithms in clinical practice.

## 2. Materials and Methods

### 2.1. Search Strategy

To conduct this review, literature searches for free text and MeSH terms were performed using several search engines: Medline (PubMed), Web of Science, Scopus, and Google Scholar. The search engines were used to find studies that focused on the application of AI in orthodontics. The last search date was 20 December 2023. For Google Scholar, the search was restricted to the first 100 most relevant articles published over the last 10 years. The search was preceded by a presearch to find the best search terms. The keywords used in the search strategy were as follows: “artificial intelligence”, “orthodontics”, “deep learning”, “neural networks”, “automatic detection”, “automated”, “caries”, “periapical lesion”, “periapical lucency”, “CBCT”, “vertebral maturation”, “skeletal age assessment”, “temporomandibular joint”, “temporomandibular joint disorders”, “osteoarthritis”, “extraction decision making”, and “cephalometric landmarks identification”. The cited articles explored the subject of AI applications in orthodontics and dentistry: dental diagnostics, cephalometric analysis, TMJ evaluation, determination of skeletal age, and treatment planning.

### 2.2. Eligibility Criteria

The following inclusion criteria were employed for this review: (1) a randomized clinical trial (RCT), (2) a cohort study, (3) a case–control study, (4) articles published in the last 10 years, and (5) articles published in English.

The following exclusion criteria were applied: (1) case reports; (2) abstracts and author debates or editorials; and (3) papers not related to practical implementations of AI programs in dentistry, particularly in orthodontics.

### 2.3. Data Extraction

Titles and abstracts were independently selected by two authors (NK and WK) following the inclusion criteria. The full text of each identified article was then analyzed to verify whether the article was suitable for inclusion. Whenever disagreement occurred, it was resolved by discussion with the third author (JJO). The authorship, year of publication, type of each eligible study, and relevance of the study for the application of AI in orthodontics were extracted by one author (NK) and examined by another author (WK).

## 3. Results and Discussion

There were 509 potential articles identified. After the removal of 183 duplicates, 226 titles and abstracts were assessed. Then, 89 papers were excluded because they did not meet the inclusion criteria and were not related to the topic of this review. All the remaining 139 papers were retrieved and analyzed to conduct this review.

### 3.1. AI Categories

**Figure 1:**
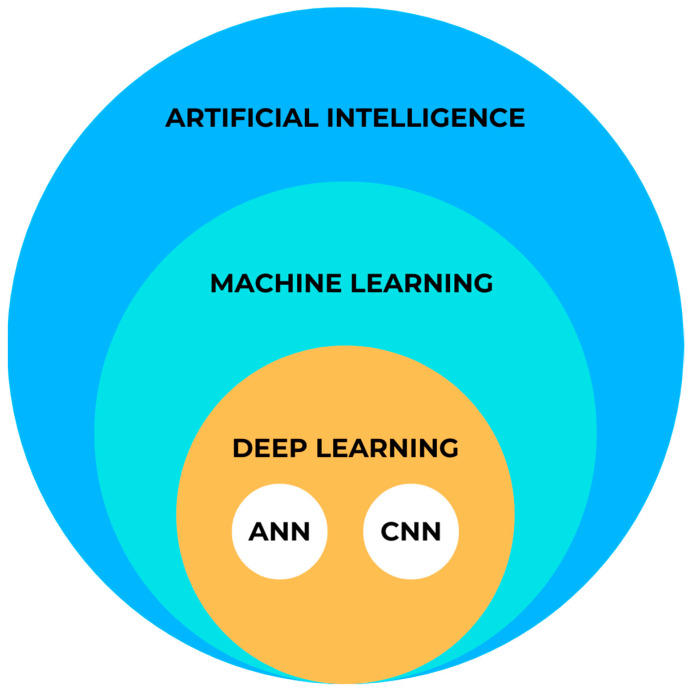
Simplified AI diagram.

AI can be classified into two main categories: symbolic AI and machine learning (ML) [17]. Symbolic AI involves structuring an algorithm in a way that is easily understandable to humans. This approach, known as Good Old-Fashioned AI (GOFAI), was dominant in AI research until the late 1980s. Symbolic AI is still useful when problems have limited outcomes, computational power is limited, or human interpretability is important. However, in healthcare, the efficiency of the GOFAI is low due to the complexity of the problems, multiple variables, and limited sets of rules [18]. Therefore, advancements in technology and computer sciences have led to the emergence of more powerful iterations of AI that are replacing the GOFAI in medical applications. Figure 1 provides a schematic representation of AI.

#### 3.1.1. Machine Learning

Machine learning (ML) is the predominant paradigm in the field of AI. Coined by Arthur Samuel in 1952, ML differs from symbolic AI because it relies on models learned from examples rather than predefined rules set by humans [19]. By utilizing statistical and probabilistic techniques, machines can enhance their performance by learning from previous models and adapting their actions when new data are introduced. This can involve making predictions, identifying new patterns, or classifying new data.

ML methods can be categorized into three types based on the learning approach and desired outcome. The first type is supervised learning, which is used for classification or prediction tasks where the outcome is already known. In this case, the algorithm learns from a labeled dataset and generalizes its knowledge to make accurate predictions with respect to unseen data. The second type is unsupervised learning, which aims to discover hidden patterns and structures in data without any prior knowledge of the outcome. This type of learning is useful for tasks such as clustering and anomaly detection. Finally, reinforcement learning involves a machine in the development of an algorithm that maximizes a predefined reward based on previous versions of the machine. This type of learning is often used in scenarios where an agent interacts with an environment and learns through trial and error [20].

#### 3.1.2. Deep Learning

Deep learning (DL) is a subset of ML that involves machines independently computing specific characteristics of an input. DL builds upon artificial neural networks (ANNs) developed in the 1990s. Recent advancements in computational technology have allowed researchers to construct more complex neural networks, referred to as “deeper” networks, to handle increasingly challenging tasks. In the field of medical imaging, DL algorithms predominantly utilize convolutional neural networks (CNNs) with high diagnostic accuracy [21,22,23].

DL differs from traditional ML methods because it enables machines to automatically extract relevant features from input data. Unlike traditional ML, DL models do not rely on human engineers to manually point these features. DL algorithms can learn and identify patterns directly from raw data, eliminating the need for time-consuming feature identification and extraction [23]. This capability has proven particularly valuable in imaging, where DL tools have shown superior diagnostic accuracy compared to experienced readers [21,24,25]. However, DL is not limited to image analysis tasks. It has shown promise in various other applications, such as medical disease diagnosis and personalized treatment recommendation [26,27,28,29].

### 3.2. AI Applications in Orthodontics

#### 3.2.1. Dental Diagnostics

The use of medical imaging methods is essential in dental patient care because they aid in the clinical diagnosis of pathologies related to teeth and their surrounding structures [30,31,32]. Radiological methods, such as orthopantomograms (OPGs) and cone-beam computed tomography (CBCT), play crucial roles in orthodontic diagnosis, treatment planning, and monitoring [33,34,35]. However, with the increasing number of radiological examinations being performed [36], there is a need for a comprehensive tool to support the process of radiological diagnosis. In response to this demand, multimodular diagnostic systems based on AI have emerged.

One such AI-based system, developed by Diagnocat Ltd. (San Francisco, CA, USA), utilizes CNNs and provides precise and comprehensive dental diagnostics. The system enables tooth segmentation and enumeration, oral pathology diagnosis (including periapical lesions and caries), and volumetric assessment. Several scientific papers have validated the diagnostic performance of this program, demonstrating its high efficacy and accuracy [37,38,39,40,41]. A study by Orhan et al. [37] reported that the AI system achieved 92.8% accuracy in the detection of periapical lesions in CBCT images and showed no statistically significant difference in volumetric measurements compared to manual methods. Similarly, a study evaluating the diagnostic accuracy of the program for periapical lesion detection on periapical radiographs (PRs) yielded comparable results [38]. However, conflicting results have also been reported, particularly regarding the accuracy of AI in the assessment of periapical lesions in OPGs [42].

In a recent study by Ezhov (2021) [43], the overall diagnostic performance of two groups, one aided by AI and the other unaided, was compared in oral CBCT evaluation. The AI system used in this study included modules for tooth and jaw segmentation, tooth localization and enumeration, periodontitis, caries, and periapical lesion detection. The results showed that the AI system significantly improved the diagnostic capabilities of dentists, with higher sensitivity and specificity values observed in the AI-aided group than in the unaided group (sensitivity: 0.8537 vs. 0.7672; specificity: 0.9672 vs. 0.9616).

**Figure 2:**
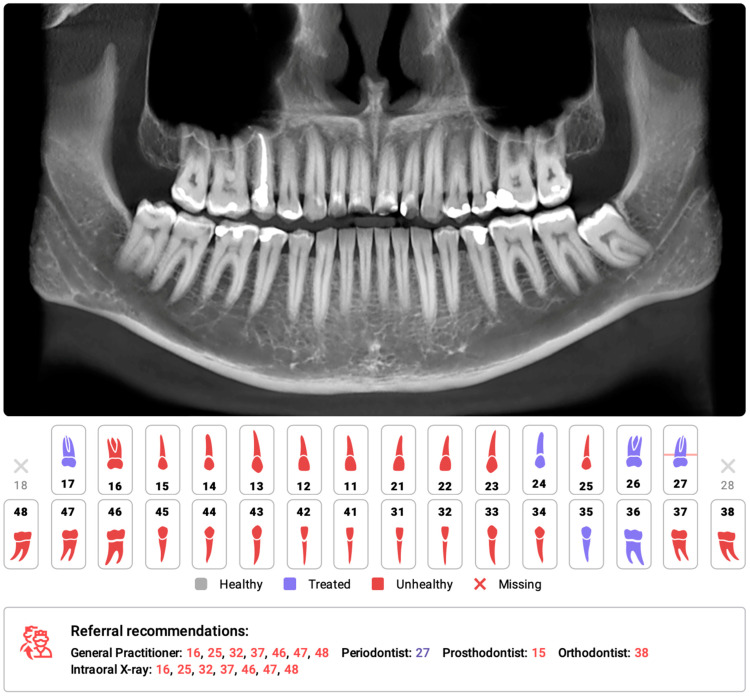
Part of the automatic diagnostic report from a CBCT scan was obtained prior to orthodontic treatment on a 24-year-old male. The software automatically identified the absence of teeth 18 and 28, as well as changes in the remaining teeth, primarily consisting of attrition and the presence of dental fillings. The program has recommended further consultations as necessary.

Several systematic reviews and meta-analyses have been conducted on the utilization of AI for identifying caries and periapical lucencies [44,45,46,47,48,49,50,51,52,53,54,55]. In a recent comprehensive study by Rahimi [54], the accuracy of classification models for caries detection was evaluated across 48 studies. The reported diagnostic accuracy varied significantly based on the imaging modality, ranging from 68% to 99.2%. The diagnostic odds ratio, which indicates the effectiveness of the test, also varied greatly from 2.27 to 32,767 across studies. The study concluded that deep learning models show promise for caries detection and may aid clinical workflows. One of the earliest meta-analyses conducted in 2019 on the computer-aided detection of radiolucent lesions in the maxillofacial region [46] yielded a pooled accuracy estimate of 88.75% (95% CI = 85.19–92.30); however, only four studies were included. A more recent meta-analysis by Sadr [52] included 18 studies and revealed that the pooled sensitivity and specificity were 0.925 (95% CI, 0.862–0.960) and 0.852 (95% CI, 0.810–0.885), respectively. The authors concluded that deep learning showed highly accurate results in detecting periapical radiolucent lesions in dental radiographs. These findings suggest that multimodal AI programs may serve as first-line diagnostic aids and decision support systems, improving patient care at multiple levels. Figure 2 shows a sample of the Diagnocat report.

#### 3.2.2. Cephalometric Analysis

Cephalometric analysis (CA) is an important diagnostic tool in orthodontics that has been in use since 1931 [56]. Over the years, advancements in technology have revolutionized CA by replacing manual assessments with digital software. This approach simplifies the measurement process and provides an automatic display of the analysis results. Automated CA has been shown to be more stable and repeatable than manual analyses, which rely heavily on operator-dependent landmark identification and often exhibit significant variability [57,58,59,60]. Accurate and repeatable landmark identification is crucial for reliable CA outcomes. Several studies have demonstrated the effectiveness of AI in identifying cephalometric landmarks. Although lateral radiography remains the most commonly used method in CA, recent AI advancements have sparked renewed interest in the use of cone-beam computed tomography (CBCT) [61].

**Figure 3:**
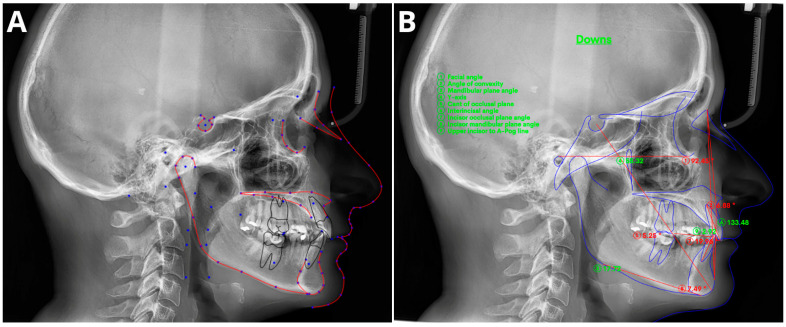
Sample of automatic cephalometric landmark tracings performed using CephX (**A**) and WebCeph (**B**) on an 18-year-old male. The results of Downs cephalometric analysis superimposed on tracings (**B**). Measurements outside the standard range marked in red and with asterix *.

The effectiveness of AI in identifying cephalometric landmarks has been studied since 1998 [62]. Numerous studies have used various automated methods and have consistently achieved high accuracy in landmark identification [59,60,63,64,65,66,67,68,69,70,71,72]. A recent study by Hwang et al. (2020) [60] concluded that automated cephalometric landmark identification can be as reliable as an experienced human reader. Similarly, Kim et al. [65], Lee et al. [71], and Dobratulin et al. [63] achieved landmark definition accuracies between 88% and 92% using AI. These authors also found that, compared with manual methods, AI methods demonstrated greater accuracy in landmark identification and reduced the time and human labor required. In other studies conducted by Hwang et al. [59] and Yu et al. [70], the authors found no statistically significant differences between the results of automated cephalometric analysis and those calculated via manually identified landmarks. Additionally, AI has been shown to significantly improve the workflow of practices, reducing analysis time by up to 80 times compared to manual analysis [72]. Figure 3 shows the definitions of the sampled cephalometric landmarks.

The utilization of CBCT in CA was first reported in the 2000s [73], but its use has remained limited due to inefficiency and time constraints. However, recent advancements in AI have revived interest in CBCT-based CA. Several studies [74,75,76,77,78,79,80,81] have shown that AI techniques are accurate and efficient for automatically identifying and analyzing landmarks, surpassing manual approaches. Kim et al. [80] found that the repeatability of artificial neural networks was higher than achieved by human reades, while Muraev et al. [81] reported that artificial neural networks (ANNs) performed as well as or better than inexperienced readers in identifying landmarks. However, Bao et al. (2023) [82] recently revealed that manual tracing is still necessary to increase the accuracy of automated AI analysis, indicating the importance of manual supervision.

Meta-analyses have generally shown high accuracy in identifying cephalometric landmarks [83,84,85,86,87,88,89]. However, the results are strongly dependent on predefined thresholds, with lower accuracies reported at a 2 mm threshold [83,85,88]. Serafin et al. [89] conducted a study in 2023 and reported a mean difference of 2.44 mm between three-dimensional (3D) automated and manual landmarking. A meta-regression analysis indicated a significant association between publication year and mean error, suggesting that recent advances in deep learning (DL) algorithms have significantly improved landmark annotation accuracy. Overall, AI tools have shown promising results in automated cephalometric analyses, but caution is advised due to potential biases in evaluated studies [83,84,85,87].

#### 3.2.3. Determination of Skeletal Age

Growth and maturation play crucial roles in orthodontics because they directly impact the effectiveness of orthodontic treatments, which are often timed to coincide with periods of rapid growth and developmental changes in facial structure. Previous studies have demonstrated that tailoring treatments to align with the patient’s growth phases can enhance treatment outcomes [90,91]. Additionally, some studies suggest that dental maturation is linked to the patient’s skeletal class [92]. Accurately assessing the rate of growth and stage of facial development is crucial in orthodontic treatment to achieve long-term results and minimize post-treatment changes caused by ongoing facial growth [93]. However, growth dynamics during adolescence differ greatly among individuals, making it insufficient to rely solely on chronological age for estimating the amount of remaining growth [94,95].

Skeletal age, which can be assessed using cervical vertebral maturation (CVM) or wrist X-rays, is a more suitable parameter for evaluating individual growth [90,96,97,98,99]. While wrist X-rays are contraindicated in standard diagnostic orthodontic routines, the CVM can be assessed using lateral cephalometric X-rays [33]. In recent years, there has been a growing body of scientific evidence supporting the diagnostic accuracy and effectiveness of AI in assessing skeletal age based on both wrist X-rays and CVM [100,101,102,103,104,105]. Despite the proven diagnostic accuracy of AI in skeletal age assessment, particularly with wrist X-rays and even index finger X-rays, concerns remain regarding the accuracy of CVM-based models [106,107]. Studies on this topic have yielded varied results, with agreement rates with human observers ranging from 58% to more than 90% [107,108,109,110,111,112]. Seo et al. (2021) reported that CNN-based models achieved more than 90% accuracy in CVM assessments, suggesting that automatic diagnosis using lateral cephalometric radiographs can accurately determine skeletal maturity [109]. However, exercise caution is important when evaluating the results of AI in CVM assessments. Other studies have reported notable discrepancies, particularly during crucial orthodontic treatment stages around the growth peak, when accuracy tends to decrease [95,110].

Caution is advised when interpreting AI-assisted CVM assessment studies due to the limited number of expert readers used to establish the gold standard for evaluation. Errors made by these readers may have influenced the study results and subsequently impacted the performance of the AI algorithm [104]. The lack of scientific evidence in meta-analyses highlights the need for a broader examination of the role of AI in CMR. A recent systematic review by The Angle Orthodontist reported that the model accuracy for test data ranged from 50% to more than 90%. The authors emphasized the importance of conducting new studies to develop robust models and reference standards that can be applied to external datasets. While these findings are encouraging, we anticipate that future advancements in AI technology will enhance the diagnostic accuracy of CMR tools, potentially making them comparable to wrist X-ray assessments for skeletal maturity.

#### 3.2.4. TMJ Evaluation

Osteoarthritis (OA) is a condition that affects joints and is characterized by the gradual deterioration of joint cartilage, bone remodeling, and the formation of osteoproliferative bodies [113]. Temporomandibular joint osteoarthritis (TMJOA) is a specific type of temporomandibular disorder that can cause significant joint pain, dysfunction, and dental malocclusion and a decrease in overall quality of life [113]. The examination of TMJ function and morphology is crucial in orthodontic and dental treatments [114]. TMJOA is one of the causes of malocclusion and facial asymmetry [115,116]. Radiographic examination, such as OPG/CBCT, confirms the presence of TMJOA by revealing bony changes [117], while MRI is the preferred modality for evaluating joint discs [114].

Recent studies have demonstrated the high diagnostic performance of AI in detecting and staging TMJOA [117,118,119,120,121]. These studies have shown the potential for automated, detailed assessment of joint morphology using various imaging techniques, including OPG, CBCT, and MRI. Therefore, the authors anticipate that the use of AI systems for TMJ diagnostic imaging will contribute to future research on early detection and personalized treatments for OA.

The few reviews and meta-analyses conducted on this topic showed the overall moderate-to-good accuracy of the tested models in TMJOA detection [122,123,124,125,126]. The 2023 study by Almasan [123] showed that the pooled sensitivity and specificity of AI in panoramic radiograph TMJOA detection accounted for 0.76 (95% CI 0.35–0.95) and 0.79 (95% CI 0.75–0.83), respectively. Similar results related to this topic were reported by Xu [126], who reported a pooled sensitivity, specificity, and area under the curve (AUC) of 80%, 90%, and 92%, respectively. A more comprehensive study carried out by Jha et al. [125] analyzed 17 articles for the automated diagnosis of masticatory muscle disorders, TMJ osteoarthrosis, internal derangement, and disc perforation. The results of the meta-analysis showed the high diagnostic accuracy of the tested AI models, with accuracy and specificity ranging from 84% to 99.9% and 73% to 100%, respectively.

#### 3.2.5. Extraction Decision Making

One of the most challenging issues during orthodontic treatment is deciding whether extraction is mandatory in a particular case. A variety of factors associated with the identified orthodontic defect, patient preferences, expected outcomes, sociocultural factors, and the professional position of the orthodontist influence the patient’s attitude toward the proposed orthodontic extraction therapy [127,128,129]. On the other hand, decisions related to extractions are influenced by the experience, training, and philosophy of the orthodontist [130,131,132,133]. All these factors render the extraction decision during orthodontic treatment very challenging, even for an experienced practitioner. Furthermore, conclusions regarding the treatment undertaken can greatly vary among experts, especially in borderline cases [134,135,136,137].

Several AI tools have been introduced in recent years to support therapeutic decision making in orthodontics [94,138,139]. Initial studies on extraction decision aids have shown promising results, with AI algorithms achieving over 80% agreement with expert decisions [140,141,142,143,144]. Xie’s study (2010) [144] revealed an 80% concurrence in extraction decisions between AI and experts, although only 20 cases were analyzed. Jung and King evaluated an ANN system [142], which achieved a 93% success rate in diagnosing extraction versus nonextraction cases based on 12 cephalometric variables and an 84% success rate for the detailed diagnosis of specific extraction patterns.

Similar results were achieved by Li et al. (2019) [143], who reported a 94% accuracy for extraction versus nonextraction predictions, 84.2% for extraction patterns, and 92.8% for anchorage patterns. These studies identified several features that are important in predicting treatment efficacy, such as crowding of the upper arch, position of anterior teeth, lower incisor inclination, overjet, overbite, and capability for lip closure. However, it is important to note that these studies have significant limitations that may introduce bias. For instance, the AI systems were trained using examples provided by a limited number of experts, which may reflect the treatment philosophies of those experts without considering the validity of those approaches. Additionally, important dental findings, such as large dental fillings, periapical lesions, periodontal damage, previous endodontic treatment, and missing teeth, were not considered [129,141,142,143,144].

Given these limitations, it is important to acknowledge that making a definitive decision on whether to proceed with orthodontic extraction therapy is often challenging, especially in borderline cases. Clinicians need to carefully evaluate the advantages and disadvantages of each treatment approach and consider the overall clinical situation. Additionally, the use of extraction decision-making tools in clinical practice carries the risk of being influenced by a specific treatment philosophy, which could impact patient care. Practitioners should strive to develop individualized treatment plans for their patients and not be influenced by rigid treatment “philosophies” [128].

#### 3.2.6. Orthognathic Surgery Decision Making and Planning

Despite significant developments in orthodontics and surgery, there is a lack of clearly established criteria for qualifying patients for surgical procedures. This issue becomes particularly problematic in borderline cases, where the orthodontist faces the decision of whether to refer the patient for surgical treatment or camouflage treatment [145,146]. The primary issue that determines the further fate of a patient is the identification of patients who benefit from orthognathic surgery. The diagnosis of a surgical case is usually confirmed via lateral cephalograms, which are the primary method for assessing sagittal skeletal deformities. The effectiveness of both the AI and ML algorithms has already been proven to identify orthognathic surgery diagnoses with over 90% accuracy [117,147,148,149,150]. One interesting study carried out by Jeong et al. [151] evaluated soft tissue profiles based on facial photographs. The evaluated CNN yielded 89% accuracy in correctly classifying surgical cases [151].

There is a limited yet promising body of literature that assesses the performance of AI in orthognathic surgery treatment planning [140,152,153]. Knoops et al. conducted a study in which they applied a 3D morphable model (3DMM) to automatically diagnose patients, categorize their risk levels, and generate simulations for orthognathic surgery treatment plans [153]. This approach achieved a sensitivity of 95.5% and a specificity of 95.2%, with an average accuracy of 1.1 ± 0.3 mm. Additionally, the positive and negative predictive values were 87.5% and 98.3%, respectively [153]. Chung et al. proposed a technique for the automatic alignment of CBCT images with optically scanned models using a DeepPose regression neural network [152]. This method surpassed the accuracy of previously top-performing techniques by 33.09% [152]. In a previously mentioned study by Choi et al., a model accurately predicted the need for surgery and provided an extraction plan for surgical patients, achieving an accuracy ranging from 88% to 97% [140].

However, it is important to mention that the research discussed above is subject to the same limitations mentioned in the chapter on extraction decision making. These limitations stem from the lack of strict guidelines, which results in a heavy reliance on expert decisions and opinions. A systematic review conducted by Smith et al. [154] further supports this notion by indicating that the results of current studies cannot be easily generalized due to their significant heterogeneity. The authors carefully concluded that AI might be a useful tool in planning orthognathic surgery. However, additional studies are needed.

It is worth noting that the abovementioned research shares the same limitations as those discussed in the extraction decision-making chapter, primarily due to the absence of clear guidelines. This leads to a heavy dependence on expert decisions and opinions. A systematic review by Smith et al. [154] further highlighted the significant heterogeneity among current studies and the difficulty in generalizing their results. The authors cautiously concluded that AI could be a valuable tool in orthognathic surgery planning, but further research is necessary.

#### 3.2.7. Treatment Outcome Prediction

Orthodontists face the challenge of selecting the most appropriate treatment strategy for each patient based on their individual expectations, socioeconomic conditions, cultural background, and skills. However, procedures such as extractions and orthognathic surgeries are irreversible and can result in permanent patient dissatisfaction. Therefore, accurately predicting treatment outcomes is crucial for both practitioners and patients. Fortunately, a growing body of literature demonstrates the effectiveness of AI in predicting orthodontic and orthognathic treatment outcomes [155,156,157,158,159,160,161,162,163].

The model proposed by Park et al. [161] achieved high accuracy in predicting treatment outcomes for Class II patients, with a mean error of 1.79 ± 1.77 mm. In a recent study by Tanikawa et al. [163], a DL model was used to predict the 3D outcomes of orthodontic and orthognathic treatment in Japanese patients, resulting in mean errors of 0.69 ± 0.28 mm and 0.94 ± 0.43 mm for the orthodontic and surgical patient groups, respectively. Similarly, Park et al. [160] evaluated a DL algorithm that accurately predicted treatment outcomes in terms of 3D facial changes, with a mean prediction error of 1.2 ± 1.01 mm. Other studies have also achieved high accuracies in predicting facial symmetry in orthognathic patients [156,158] and 3D facial soft tissue changes in cleft patients after orthognathic surgery [164].

In addition to treatment outcomes, AI has also been used to predict patients’ experiences during clear aligner treatment. Xu et al. [159] developed a system that achieved close to 90% prediction accuracy in predicting patients’ pain, anxiety, and quality of life. This study highlights the importance of considering patient experience and shifting the focus from solely cosmetic or functional outcomes.

A recent scoping review [165] revealed that AI models are not only efficient but also outperform conventional methods in orthognathic treatment planning and outcome prediction. This review highlighted the reliability and reproducibility of these models, suggesting their potential to improve clinical outcomes, especially for less experienced practitioners. However, a recent meta-analysis [166] emphasized the need for caution and restraint when adopting AI advancements in orthodontics.

#### 3.2.8. Patient Monitoring

The COVID-19 pandemic has brought attention to the importance of social distancing, remote work, and telemedicine [167]. Orthodontic treatment typically lasts approximately 20 months [168] and requires regular progress monitoring and potential complications. Traditional methods of monitoring can be time-consuming and repetitive. However, recent advancements in orthodontics, such as self-ligating systems and aligners, along with the implementation of telemedicine, have led to the development of dental monitoring (DM) [169].

The DM system consists of three integrated platforms: a mobile app for patients, a web-based Doctor Dashboard^®^, and a movement-tracking algorithm that analyzes pictures taken by the patient. The goal of DM is to reduce in-office visits, detect aligner incidents and misfitting, and personalize treatment for each patient [170]. Several studies have already demonstrated the value of DM in orthodontic treatment, including reducing chairside time, improving patient compliance [171,172], early detection of orthodontic emergencies [173], reducing orthodontic relapse [174], remote monitoring of aligner fit [169,174,175], and improving oral hygiene status [176]. Interestingly, Homsi et al. [177] reported that digitally reconstructed models obtained remotely were as accurate as those obtained through intraoral scans.

However, the implementation of AI in remote care for orthodontic patients is still an underexplored topic with limited evidence. A recent systematic review [178] showed that DM showed promise in improving aligner fit and reducing the number of in-office visits during ongoing orthodontic therapy. These findings emphasize the need for further research in this area.

### 3.3. Implementation Considerations

While the potential of AI to improve patient management in orthodontics is vast, its impact has been proven in only a limited number of cases. Most of the literature on this subject consists of retrospective studies without support from large randomized controlled trials. However, we might expect such studies in the coming years due to the exciting nature of this topic and the increasing supply of AI solutions. Financial investments and the number of introduced AI technologies are rapidly growing; in 2022, there were 69 new FDA-approved products associated with USD 4.8 billion in funding. By 2035, product-year funding is projected to reach USD 30.8 billion, resulting in 350 new AI products [8].

Despite many optimistic studies demonstrating the high performance of AI algorithms in a variety of tasks, the further incorporation of AI algorithms into everyday clinical practice remains a matter for the future. Most of the aforementioned programs were introduced within the past 2–3 years. On average, 17 years are required for medical innovations to be implemented in clinical practice [179,180]. The process of implementing AI in workflows and clinical practice requires meeting a number of requirements to ensure sufficient clinical quality and patient safety. As indicated by Pianykh [9], there are still important issues to overcome. The first issue is the lack of reproducibility. AI models are typically developed using limited and specific datasets, which makes it challenging for them to perform well on a wide range of data. The second issue is the lack of adaptivity. Existing AI models are not designed to constantly adjust to changes in their environment. The third issue is the absence of robust quality control mechanisms for AI, which increases susceptibility to data errors, outliers, and sudden shifts in trends. Finally, there is a lack of integration between AI algorithms and workflows, which prevents them from effectively adapting to changes in the data environment. To address these issues, continuous learning AI needs to be developed. This approach enables the AI tool to adapt continuously to changes in the data [9]. With continuous learning, AI algorithms can make live adjustments, preventing performance deterioration over time. Like in any technology used in medicine, there is a need for a sufficient AI governance process to maintain the quality of results and ensure patient safety [181,182]. The need for continuous evaluation of algorithm quality should be kept in mind to prevent degradation in performance and to allow appropriate early intervention. Moreover, privacy issues, safety concerns, and health inequities (such as AI algorithms that exacerbate racial or income disparities) are a few more general issues related to the application of AI in medicine, and they have recently been highlighted in The Lancet [183]. While a wide range of AI products are available, scientific evidence regarding their validation and effectiveness in general medicine and specific fields such as orthodontics remains limited [184].

Despite the availability of a wide range of products, there is still limited scientific evidence regarding the validation and effectiveness of AI products in general medicine and narrow fields such as orthodontics [184]. Despite the generally optimistic results of various AI tools, the issues highlighted above underscore the necessity of exercising considerable caution when introducing AI into daily practice.

## 4. Conclusions

Undoubtedly, AI has the potential to revolutionize medicine, particularly in the field of diagnostic imaging, including orthodontics. The continuous advancement of AI algorithms that support pretreatment diagnostic processes allows the visualization of outcomes and facilitates decision making during treatment, placing orthodontics among the disciplines benefiting the most from the introduction of AI technology. However, due to the high complexity and associated unpredictability of AI, these tools should be treated with caution, and their results should be regularly and manually validated.

## Data Availability

Not applicable.
